# Hypotensive and Cardioprotective Potential of Yellow Bedstraw Extract-Based Oral Liquid in Spontaneously Hypertensive Rats

**DOI:** 10.3390/ijms25158346

**Published:** 2024-07-30

**Authors:** Jovana Bradic, Anica Petrovic, Aleksandar Kocovic, Slobodanka Mitrovic, Vladimir Jakovljevic, Nevena Lazarevic, Sergey Bolevich, Igor Simanic

**Affiliations:** 1 Department of Pharmacy, Faculty of Medical Sciences, University of Kragujevac, 69 Svetozara Markovica St., 34000 Kragujevac, Serbia; jovanabradickg@gmail.com (J.B.); salekkg91@gmail.com (A.K.); nevenasdraginic@gmail.com (N.L.); 2 Center of Excellence for Redox Balance Research in Cardiovascular and Metabolic Disorders, 69 Svetozara Markovica St., 34000 Kragujevac, Serbia; drvladakgbg@yahoo.com; 3 Department of Pathology, Faculty of Medical Sciences, University of Kragujevac, 69 Svetozara Markovica St., 34000 Kragujevac, Serbia; smitrovic@medf.kg.ac.rs; 4 Department of Physiology, Faculty of Medical Sciences, University of Kragujevac, 69 Svetozara Markovica St., 34000 Kragujevac, Serbia; 5 Department of Human Pathology, Sechenov First Moscow State Medical University, 8 Trubetskaya Street St., 119991 Moscow, Russia; bolevich2011@yandex.ru; 6 Specialized Hospital for Rehabilitation and Orthopedic Prosthetics, Sokobanjska 17, 11000 Beograd, Serbia; dr.igorsimanic@yahoo.com; 7 Department of Physical Medicine and Rehabilitation, Faculty of Medical Sciences, University of Kragujevac, 69 Svetozara Markovica St., 34000 Kragujevac, Serbia

**Keywords:** yellow bedstraw, cardiovascular protection, hypertension, oxidative stress, antioxidant activity

## Abstract

This study aimed to prepare, characterize and assess the antioxidant activity of yellow bedstraw extracts (YBEs), focusing on identifying extracts with high antioxidant capacity. The selected extract was loaded into an oral liquid formulation and further investigated for its therapeutic potential in reducing blood pressure and associated complications in spontaneously hypertensive *Wistar kyoto* rats (SHR). Rats were divided into untreated SHR and SHR treated with a YBE-based oral formulation over four weeks. After treatment, blood pressure was measured, and cardiac function was assessed using the Langendorff technique to simulate ex vivo ischemic conditions. Prooxidant levels were assessed in plasma while antioxidant activity was evaluated in red blood cells. Histological analyses of heart, kidney, and liver samples were conducted to assess pathological changes induced by hypertension. Our results showed that the oral formulation loaded with ethanol YBE effectively reduced blood pressure, preserved myocardial function under ischemic stress, and decreased oxidative stress markers in blood. Importantly, our formulation with YBE demonstrated potential in attenuating structural kidney damage associated with hypertension. Overall, these findings suggest a cardioprotective effect of orally administered YBE formulation, highlighting its potential as an herbal supplement. However, clinical studies are warranted to validate these findings and explore the extract’s suitability for clinical use.

## 1. Introduction

Alarming data suggest that long-standing hypertension might lead to structural and functional abnormalities of the heart that profoundly impair the quality of life of patients, elevate healthcare costs, and in severe cases, may even result in mortality. It is well known that patients with uncontrolled or inadequately controlled blood pressure have a higher risk of hypertensive heart disease associated with the micro- and macroscopic remodeling of the heart, functional alterations, and adaptions of the arterial system [1]. The epidemiology data regarding this condition is quite concerning, since the number of patients with hypertension has doubled globally in the last three decades, and nowadays over 1.2 billion adults in the world have hypertension [2,3]. Moreover, about 46% of adults with hypertension are unaware of having hypertension, while only 21% of patients with the condition have controlled blood pressure [3]. It has been reported that hypertensive heart disease is associated with disturbed diastolic function, high risk of atrial fibrillation, kidney disease, etc., thus emphasizing the importance of the appropriate control of blood pressure in the prevention of ischemic heart disease. Scientists have established several mechanisms involved in this pathology, the most important are the following: oxidative stress, increased inflammatory response, the depletion of ATP, Ca^2+^ overload, and mitochondrial dysfunction [4,5]. Disturbing data indicate that approximately 47.5% of patients treated with available antihypertensive drugs do not achieve therapy goals. Moreover, those drugs have several disadvantages including limited efficacy in the reduction of complications, mainly pathological alterations to the heart and kidneys that occur in hypertensive patients. A novel approach in this area of investigation highlights non-pharmacological strategies with medicinal plants rich in polyphenols. These natural compounds have been proven to display antioxidant, anti-inflammatory, and cardioprotective effects, thus contributing to the attenuation of hypertension and damage of the main organs affected by this condition [6].

Yellow bedstraw (*Galium verum* L. or lady’s bedstraw) is a perennial plant, used in traditional medicine worldwide for different pathologies. Throughout history, its use in folk medicine relies on diuretic, choleretic, antidiarrheal, spasmolytic, sedative, anticancer, antiepileptic, and wound healing properties. Yellow bedstraw has also been shown to display beneficial effects against liver disorders and cardiovascular diseases due to its rich antioxidant phenolic and flavonoid content. Phytochemical investigations revealed that yellow bedstraw contains various bioactive compounds such as flavonoids, anthraquinones, triterpenes, iridoids, and phenolic compounds such as chlorogenic and caffeic acids exhibiting potent antioxidant properties [7,8,9]. We hypothesized that these antioxidant and anti-inflammatory compounds of yellow bedstraw might express promising effects in reducing blood pressure and mitigating cardiovascular events.

Recognizing the constraints of established therapeutic approaches, the aim of our study was to provide an innovative natural formulation based on yellow bedstraw extract (YBE) intended for hypertension control and the prevention of hypertension-induced complications. After the preparation and selection of extract with the maximal antioxidant activity, the protective potential of prepared formulations was tested in spontaneously hypertensive rats. To our best knowledge, this is the first study to provide evidence regarding the potential of an oral liquid formulation loaded with YBE to reduce blood pressure as well to protect the heart and kidney from damage in hypertensive conditions.

## 2. Results

### 2.1. Preparation and Chemical Characterization of Extract

**Figure 1:**
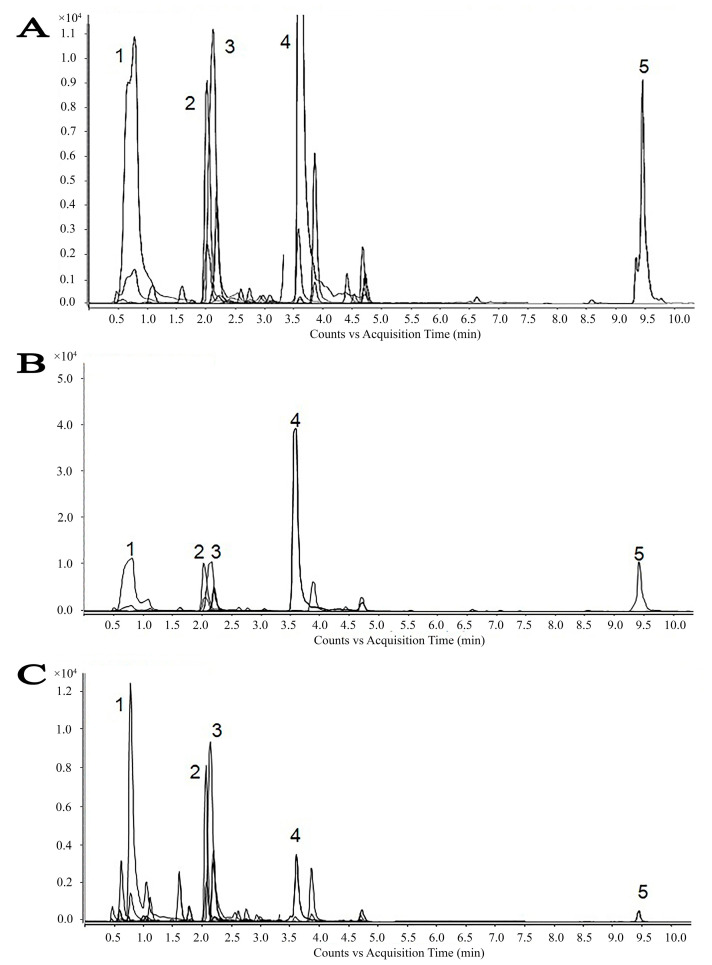
HPLC chromatograms of acetonic, ethanolic, and aqueous extracts of yellow bedstraw. 1—chlorogenic acid; 2—cynaroside; 3—isoquercetin; 4—Quercetin; 5—ursolic acid. (**A**) Chromatogram of yellow bedstraw acetonic extract; (**B**) chromatogram of yellow bedstraw ethanolic extract; and (**C**) chromatogram of yellow bedstraw aqueous extract.

**Figure 2:**
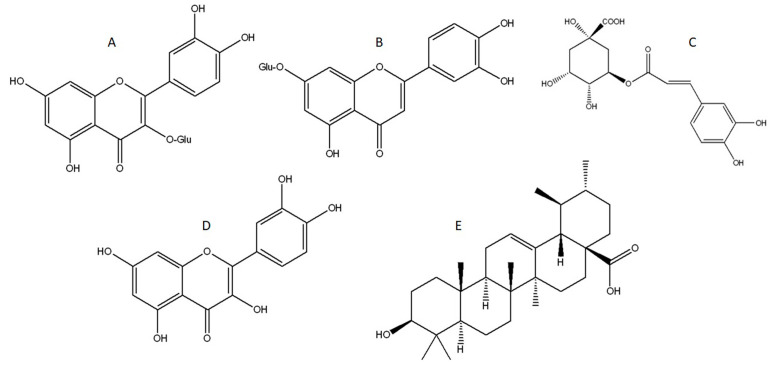
Chemical structures of compounds identified in the yellow bedstraw extracts: (**A**)—isoquercetin; (**B**)—cynaroside; (**C**)—chlorogenic acid; (**D**)—quercetin; and (**E**)—ursolic acid.

Chromatograms for the tested extracts are presented in Figure 1, while data on the retention time and content of the identified bioactive compounds are presented in Table 1. Of the 35 standards tested, 26 were identified in the analyzed samples while epigallocatechin gallate, vitexin, rutin, apiin, ellagic acid, o-coumaric acid, quercitrin, baicalin, and glycyrrhetinic acid were not detected. Liquid chromatography with a diode array detector and tandem mass spectrometry (LC–DAD–MS/MS) analysis suggested the presence of various bioactive molecules, while the most abundant compounds in all extracts were isoquercetin, cynaroside, and chlorogenic acid, while acetone and ethanol extracts contained significant amounts of quercetin and ursolic acid as well (Table 1, Figure 1). This analysis provided evidence that water, ethanol, and acetone extracted similar biomolecules, but in different quantities. Chemical structures of the main identified compounds are presented in Figure 2.

### 2.2. Polyphenol Content and Antioxidant Activity of Extracts

**Figure 3:**
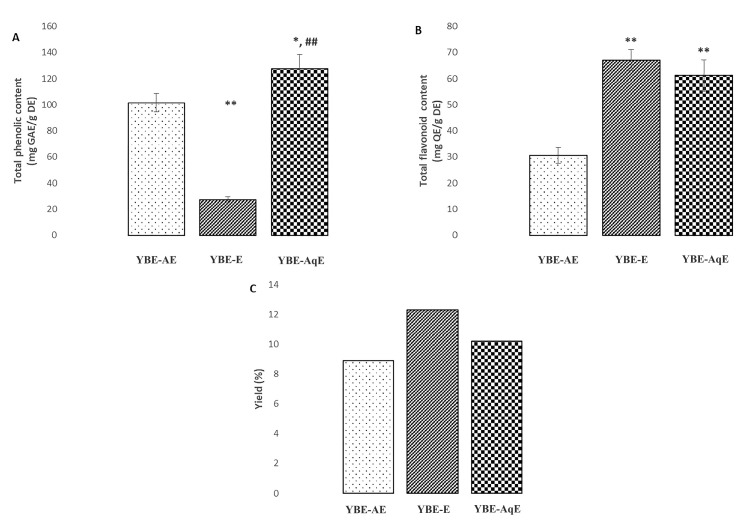
(**A**)—total phenolic content (TPC); (**B**)—total flavonoid content (TFC); and (**C**)—yield of different yellow bedstraw extracts. Values are means of three biological replicates ± SD. GAE—gallic acid equivalent; QE—quercetin equivalent; DE—dry extract; YBE-AE—yellow bedstraw acetone extract; YBE-E—yellow bedstraw ethanol extract; YBE-AqE—yellow bedstraw aqueous extract; *, statistical significance at the level of *p* < 0.05 in relation to YBE-AE group; **, statistical significance at the level of *p* < 0.01 in relation to YBE-AE group; and ##, statistical significance at the level of *p* < 0.01 in relation to YBE-E group. Data were analyzed using one-way ANOVA test.

Additionally, our findings indicate significant difference in TPC and TFC with respect to extracting solvents (Figure 3A–C). The results of TPC were as follows: aqueous (127.57 ± 11.25 mg GAE/g DE) > acetone (101.58 ± 7.96 mg GAE/g DE) > ethanol (27.34 ± 2.12 mg GAE/g DE) extract. On the other hand, the highest TFC was observed in ethanol extract (67.11 ± 4.13 mg QE/g of DE), while the lowest was observed in acetone extract (30.51 ± 3.37 mg QE/g of DE).

**Figure 4:**
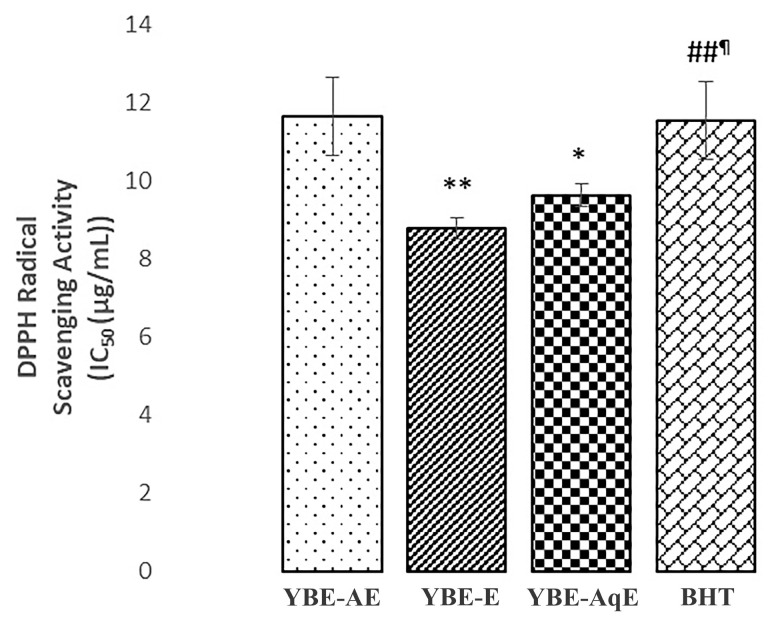
DPPH radical scavenging activity (IC_50_ (µg/mL). YBE-AE—yellow bedstraw acetone extract; YBE-E—yellow bedstraw ethanol extract; YBE-AqE—yellow bedstraw aqueous extract; and BHT—butylated hydroxytoluene. Values are means of three biological replicates ± SD; *, statistical significance at the level of *p* < 0.05 in relation to YBE-AE group; **, statistical significance at the level of *p* < 0.01 in relation to YBE-E group; ##, statistical significance at the level of *p* < 0.01 in relation to YBE-E group; and ¶, statistical significance at the level of *p* < 0.05 in relation to YBE-AqE group. Data were analyzed using a one-way ANOVA test.

The results of the DPPH assay indicate that all of the investigated extracts of yellow bedstraw possessed DPPH free-radical-scavenging activity (Figure 4). However, the highest activity was observed for ethanol extract, followed by aqueous extract, and those activities were higher in relation to the standard value (*p* < 0.05).

### 2.3. Physical Characterization, pH Value, and Microbiological Quality of Oral Liquid Formulation Based on YBE

For the preparation of YBE oral formulation, we selected ethanol extract based on its superior antioxidant properties and rich flavonoid content in comparison to acetone and water extracts. After incorporating the chosen extract into an oral liquid formulation, we conducted physical characterization, pH value, and microbiological quality testing. The YBE formulation exhibited a brown color, had a characteristic odor and was a liquid consistency after 24 h of preparation. Moreover, the pH value of the examined formulation was 5.24 ± 0.20. In order to ensure the formulation’s safety and prevent harmful effects of the formulation in consumers, we conducted a microbiological test. We revealed no contamination with *E. coli* and the fungal/yeast count was below 8 CFU/g. The total viable aerobic count complied with specifications for the formulation (not more than 10 CFU/g).

### 2.4. Cardioprotective Effects of YBE-Based Oral Liquid in Rats

Unleashing the potential of the YBE-based oral liquid in promoting cardiovascular health involved an assessment of the impact of the chronic consumption of this natural formulation on blood pressure and heart rate as well as ex vivo ischemic heart damage.

#### 2.4.1. Effects of YBE-Based Oral Liquid on Blood Pressure and Heart Rate

Our results suggested that systolic and diastolic blood pressure (SBP and DBP), as well as heart rate (HR), measured through the use of the tail-cuff method were decreased in rats treated with YBE oral liquid compared to untreated spontaneously hypertensive rats (Table 2).

#### 2.4.2. Effects of YBE-Based Oral Liquid Formulation on Heart Function

After obtaining the abovementioned results, we continued the second part of our investigation where we evaluated the effect of a yellow bedstraw ethanol extract-based oral liquid on myocardial injury by using a Langendorff model of ischemia–reperfusion or hypoxia–reoxygenation.

**Figure 5:**
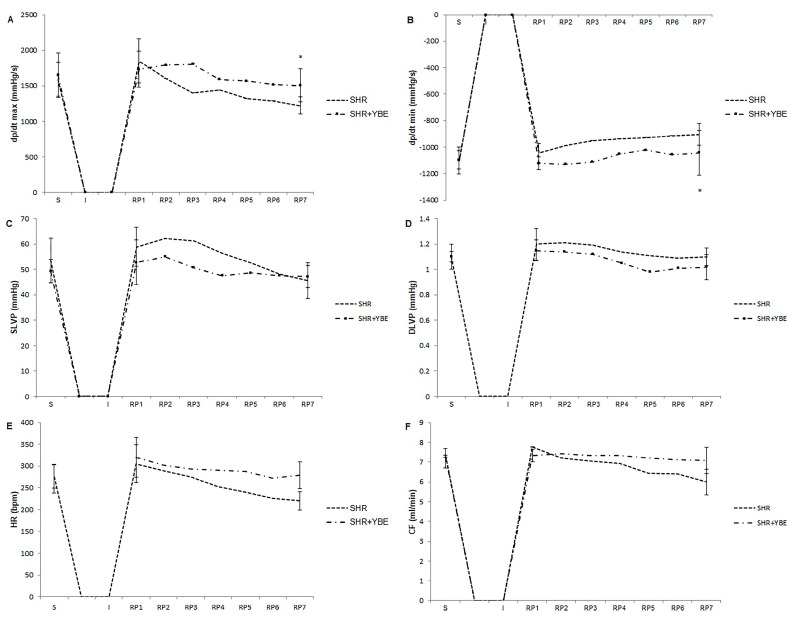
Effects of treatment with oral formulation loaded with YBE on ex vivo cardiac function in spontaneously hypertensive rats. (**A**)—dp/dtmax; (**B**)—dp/dtmin; (**C**)—SLVP; (**D**)—DLVP; (**E**)—HR; and (**F**)—CF. Data are presented as mean values ± standard deviation (X ± SD). *, statistical significance *p* < 0.05 between SHR and SHR + YBE; S—stabilization; I—ischemia; R1—first minute of reperfusion; R7—last, 30th minute of reperfusion; SHR—spontaneously hypertensive rats; YBE—yellow bedstraw extract; and *n* = 10 per group. Data were analyzed using a one-way analysis of variance (ANOVA).

Firstly, we focused on determining the effect of a YBE oral liquid formula as a preconditioning agent on parameters of heart function, such as dp/dt max, dp/dt min, SLVP, DLVP, HR, and CF. It was noticed that all of the measured parameters were impaired after a 20 min period of ischemia in the hypertensive untreated rats, thus confirming the harmful impact of myocardial ischemia. Values of dp/dt max were significantly higher in animals treated with the YBE oral liquid in comparison to SHR untreated rats at the end of the reperfusion period (Figure 5A). Additionally, values of dp/dt min parameter were significantly decreased at the end of reperfusion (R7) in SHR + YBE compared to the SHR control (Figure 5B). The chronic administration of our natural herbal formulation led to both preservation and improvement in the contractile power of the heart through a significant increase in dp/dt max and dp/dt min at the last point of the reperfusion period compared to prior values, before ischemia. Beneficial effects of the YBE formulation were also reflected in the prevention of SLVP and DLVP values from decreasing due to ischemic conditions, which can be interpreted as the capacity of YBE to preserve systolic and diastolic heart function (Figure 5C,D). In addition to the aforementioned protective effects on functional properties of the heart, treatment with YBE significantly increased heart rate values (Figure 5E) and CF in the last minute of reperfusion compared to the untreated group (Figure 5F).

#### 2.4.3. Effects of YBE Oral Liquid on Systemic Redox State

**Figure 6:**
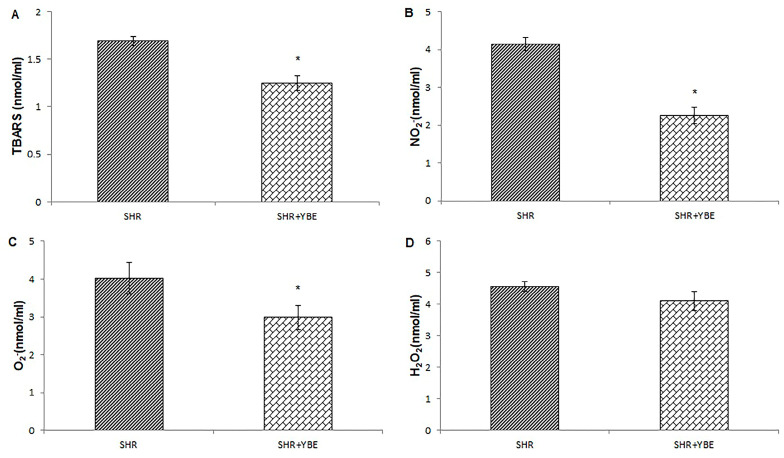
Effects of treatment with oral formulation loaded with YBE on the levels of prooxidant markers determined in blood samples of spontaneously hypertensive rats. (**A**)—TBARS; (**B**)—NO_2_^−^; (**C**)—O_2_^−^; and (**D**)—H_2_O_2_. Data are presented as mean values ± standard deviation (X ± SD); *, statistical significance *p* < 0.05 between SHR and SHR + YBE; and *n* = 10 per group. Data were analyzed using an independent samples *t*-test.

**Figure 7:**
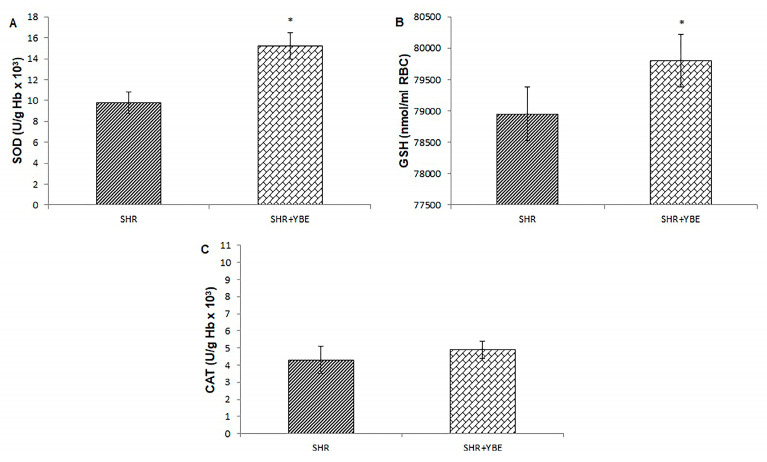
Effects of treatment with oral formulation loaded with YBE on the levels of antioxidant defense system markers determined in blood samples of spontaneously hypertensive rats; (**A**)—SOD; (**B**)—GSH^−^; (**C**)—CAT Data are presented as mean values ± standard deviation (X ± SD); *—statistical significance *p* < 0.05 between SHR and SHR + YBE; *n* = 10; Data were analyzed using an Independent Samples *t*-test.

In order to thoroughly assess the protective impact of a YBE oral liquid formulation on hypertension and associated cardiac dysfunction, we followed alterations in the systemic redox state after the chronic administration of the formulation. Our results clearly indicate that YBE liquid formulation affects the systemic redox state positively. After a 4-week treatment, a significant decrease in prooxidant markers, such as O_2_^−^, NO_2_^−^, and lipid peroxidation index (TBARS), was observed, indicating the alleviation of oxidative stress (Figure 6A–D). Moreover, the consumption of YBE oral liquid led to a rise in SOD and GSH compared to untreated SHR rats (Figure 7A–C).

### 2.5. Effects of YBE Oral Liquid on Cardiac, Kidney and Liver Tissue

**Figure 8:**
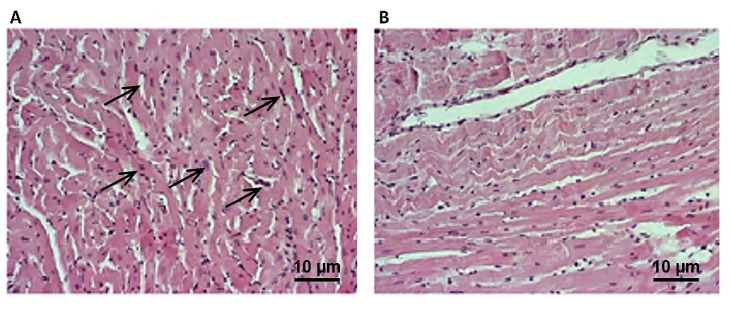
Effects of YBE oral liquid formulation on myocardium morphology—histopathological changes (magnification ×400). (**A**)—SHR group and (**B**)—SHR + YBE group. The black arrows indicate degenerative changes in myocardium of spontaneously hypertensive rats.

In untreated SHR animals, necrosis and degenerative changes were more pronounced and included zonal, confluent necrosis, with hypereosinophilia, fiber fragmentation, and the loss of nuclei (grade IV). Our results highlighted the alleviation of cardiac degenerative changes after 4-week treatment with YBE oral liquid formulation, which confirms its potency to preserve myocardial structure in hypoxic conditions (grade II) (Figure 8).

**Figure 9:**
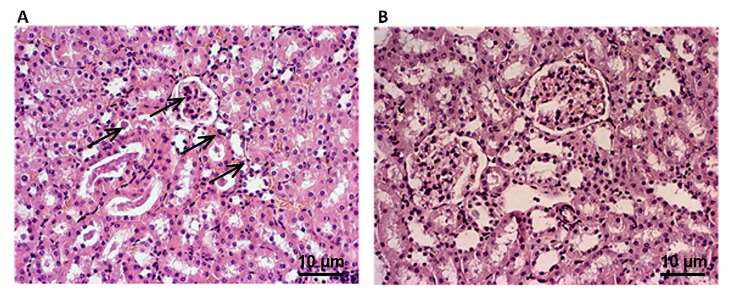
Effects of YBE oral liquid formulation on kidney morphology–histopathological changes (magnification ×400). (**A**)—SHR group and (**B**)—SHR + YBE group. The black arrows indicate degenerative changes in kidney of spontaneously hypertensive rats.

In addition, we conducted brief assessments of kidney and liver morphology to confirm the safety of our formulation. Our results indicate that the YBE liquid formula has no nephrotoxic or hepatotoxic potential. Wall thickening with subsequent lumen narrowing was observed in the kidneys of SHR rats (grade III), especially in the arterioles and small muscular arteries. The glomeruli were generally of regular morphologies, with focal changes in the form of hypocellularity, the enlargement of the subcapsular space, and occasional eosinophilic homogenization similar to fibrosis. The most significant changes were present in the proximal tubules. According to the previously defined score, histological changes in the kidneys were evaluated and found to be more pronounced in untreated rats with damage affecting 50 to 75% of the surface (Figure 9). Moreover, kidney damage was slightly decreased after YBE oral liquid treatment in SHR rats (grade II).

**Figure 10:**
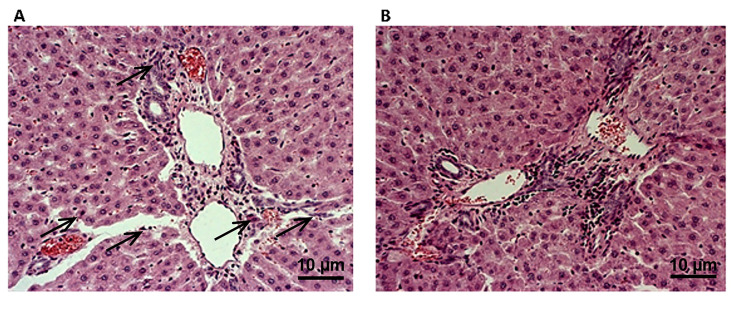
Effects of YBE oral liquid formulation on liver morphology–histopathological changes (magnification ×400). (**A**)—SHR group and (**B**)—SHR + YBE group. The black arrows indicate degenerative changes in liver of spontaneously hypertensive rats.

In order to reveal if 4-week oral liquid application may exert certain hepatotoxic effects, a histopathological analysis of liver tissue was also performed. There were no differences in histological changes in the liver between the groups after treatment with YBE. The following changes were observed in all animals: the hepatic parenchyma was a mostly preserved lobular structure with a centrally positioned vein from which, in irregularly radial arrangement, hepatocyte beds are placed, between which sinusoidal spaces are easily and perivenularly expressed as dilated, mostly normal cellularities with noticeable, single, irregularly spaced mononuclear cells and Kupffer macrophages. Additionally, port spaces were normocellular, focally containing more than one biliary duct. Without regularity, with respect to the lobular architecture of the liver, degenerative changes of the hydrops type and microfoci, mainly of unicellular necrosis, were observed. Hyperemia, most pronounced perivenularly, and a focal hydropsy degeneration of hepatocytes and necrosis were present (Figure 10).

## 3. Discussion

To address the drawbacks of current hypertensive therapy such as the necessity for multiple medications, side effects, and drug interactions, we aimed to develop and examine a natural herbal supplement that offers an alternative to conventional medications for managing hypertension. The first part of our study involved the preparation and chemical characterization of three different extracts of yellow bedstraw in order to select the most effective extract for antioxidant action. Water was chosen as the solvent since herbal teas, prepared by extracting in boiling or hot water, represent the widely used forms of plant species in traditional medicine, while ethanol and acetone have been listed as green solvents, i.e., environmentally friendly solvents [10,11]. Additionally, we wanted to analyze the in vitro antioxidant potential of these three extracts by using a DPPH test in order to see which one had the best potential for use in the second part of the study. The ethanol extract had the highest antioxidant potential, and this was partially expected since several papers support the fact that the highest antioxidant activity may be gained if ethanol is used as a solvent [12,13,14,15,16,17]. The DPPH test is recognized as an accurate and convenient assay for the evaluation of total antioxidant capacities of plant extracts. Numerous reports support the fact that the antioxidant potential of plant extracts is dedicated to the presence of phenolic compounds. In that sense, it would be expected to observe a linear correlation between the total phenolic content of yellow bedstraw and the potential of the extract to neutralize the DPPH free radical. Nevertheless, we revealed a weak antioxidant activity of the aqueous extract of lady’s bedstraw compared to the ethanolic one, although the aqueous extract showed the highest phenolic content. These findings might be explained by the fact that the method for the assessment of total phenolic content does not detect all compounds which contribute to antioxidant effects of the extract. Furthermore, the antioxidant capacity of the plant extract does not depend only on the concentration of biomolecules in the extract, but also on the structure and interaction between them [17,18]. Previous studies also emphasized the potential of ethanol extract to neutralize the DPPH radical with an IC50 of 0.136 mg/mL [19] and 105.43 μg/mL [20]. Slight variations between the DPPH potential of our extract and previously tested extracts appear to be logical since the antioxidant activity of the plant depends on the locality and the extraction process. In fact, extraction under reflux, applied in our research, has been considered as one of the most efficient techniques for the isolation of antioxidant components from plant species [21,22].

The selected ethanolic extract was used as an ingredient for oral liquid formulation development, which was further studied for in vivo cardioprotective potential. The choice of the ethanol extract was based on it having the highest flavonoid content, since flavonoids, among all compounds found in YBE, have the greatest cardioprotective potential. Additionally, the ethanol extract exhibited the highest antioxidant activity via the DPPH radical scavenging assay, which is of great importance due to a well-known benefit of antioxidants for improving cardiac health. Our data showed that treatment with YBE oral liquid formulation markedly lowered blood pressure in SHR rats. This reduction in blood pressure may be linked to the presence of valuable compounds in YBE such as ursolic and chlorogenic acid, isoquercetin, cymaroside, etc. Previous research highlighted the effects of ursolic acid in hypertension management through a decrease in the level of endothelin-1 (endothelium contracting factor) and elevated activity of endothelial nitric oxide synthase (eNOS) [6,23]. Taking into consideration that hypertension is associated with a disturbed concentration of endothelial relaxation factors, it appears logical that the promotion of NO production induced by ursolic acid leads to vasorelaxation and blood pressure control. In addition to these cardioprotective mechanisms, chlorogenic acid may contribute to blood pressure lowering through a reduction in plasma angiotensin-1-converting enzyme and consequent drop in the plasma level of angiotensin II [24]. To the best of our knowledge, this is the first study that provides evidence regarding the hypotensive effects of YBE that implies the possible role of a YBE herbal liquid for the prevention and treatment of hypertension.

It is already known that reperfusion after a prolonged period of ischemia causes most damage via the exacerbation of oxidative stress, which is a major cause of myocardial injury. Hence, a growing body of evidence supports antioxidant therapy, especially medicinal plants, as a promising non-pharmacological approach for the prevention of myocardial ischemic damage [25]. Numerous data support the fact that natural extracts rich in ursolic acid may trigger cardioprotection through the attenuation of ischemia-induced damage, the prevention of cardiac fibrosis development, slowing down atherosclerosis, etc. [23]. In line with our findings, previous research confirmed that ursolic acid can lead to an improvement in compromised systolic and diastolic function via alterations in dp/dt max and dp/dt min in diabetic rats [6]. We might assume that the presence of chlorogenic acid in our natural herbal formula also provided a valuable contribution in the alleviation of impaired left ventricular contractility [26]. Among numerous cardiac health-promoting properties, the impact on calcium homeostasis regulation is one of the mechanisms through which our developed oral liquid preserves cardiac contractility [27].

Our study’s results highlighted the potential of a YBE oral liquid formulation to reduce oxidative stress. The decrease in O_2_^−^ level can be explained via the elevation in SOD activity, an enzyme which catalyzes the formation of H_2_O_2_ using O_2_^−^ from mitochondria. Another possible explanation for the diminishing ROS activity with the YBE oral liquid formulation is the fact that polyphenols and flavonoids are proven to be the inhibitors of xhantin oxidase (XO), which contributes to the formation of H_2_O_2_ and O_2_^−^ [28]. This refers to several components of YBE, such as quercetin, diosmine, catechine, epicatechine, and isorahmnetine [6,29,30]. Several authors confirmed the antioxidant properties of YBE and its components as a free radical scavenger via the neutralization of the DPPH (2,2-Diphenyl-1-picrylhydrazyl) radical. Therefore, another way to impact oxidative stress might be via the direct scavenging property, as a mechanism independent of CAT and GSH, since their levels did not change after treatment with the oral YBE formulation [8,31,32]. In addition, triterpenoid ursolic acid, as a component of YBE, might be attributed to the antioxidant effect in myocardial damage [33]. Indeed, all of these beneficial changes in oxidative stress markers via YBE treatment may be a consequence of the synergistic action of all of the extract’s components. These findings are of great importance since oxidative stress has been linked to the development and progression of hypertension. Endothelial dysfunction has been associated with an imbalance between NO and reactive oxygen species (ROS), which results in vasoconstriction, inflammation, and other pathological processes on the vessel wall [34]. On the other hand, the strong antioxidant capacity of our developed oral liquid formulation with YBE may significantly contribute to the prevention and control of hypertension and also act protectively on endothelial function. While currently available antihypertensive drugs either slightly affect or do not affect endothelial function, our natural formula was able to not only reduce blood pressure values but also improve coronary vasodilatory response as verified by increased coronary flow, probably through elevated NO production [34].

Four-week treatment with the YBE oral liquid formulation confirmed its potency to preserve myocardial structure in hypoxic conditions according to our study. We also aimed to confirm the safety of our formulation by examining its impact on kidney and liver morphology, and proved that the YBE liquid formula has no nephrotoxic or hepatotoxic potential. The kidney damage was alleviated in YBE-treated SHR rats, which is in line with the current knowledge about flavonoids having nephroprotective potential, through the modulation of oxidative stress and inflammation [35].

The strength of our investigation is in showing the hypotensive properties of YBE for the first time and providing a deeper insight into how this phytotherapeutic can prevent cardiac damage in hypertensive conditions. A possible limitation of this study may be the fact that the cardioprotective potential of isolated compounds from YBE was not tested; however, it is more likely that positive effects on the heart come from the synergistic effects of several phenolics and flavonoids. Additionally, an examination of the time-dependent effects of the YBE oral formulation would certainly provide comprehensive evidence about its role in cardioprotection and may be a direction for future research in this field.

## 4. Materials and Methods

### 4.1. Preparation of Extracts

The aerial parts (stem, leaves, flowers, and fruits) of yellow bedstraw were collected in July 2023 in the village Dobroselica, on the southern cliff of the mountain Zlatibor (GPS coordinates: 43°42′59.99″ N and 19°41′59.99″ E). Voucher specimens were deposited at the Institute of Botany and Jevremovac Botanical Garden, Faculty of Biology, University of Belgrade (BEOU 17417). The collected material was dried under the shade, ground to a powder and passed through a sieve with 0.75 mm holes (sieve 0.75). A total of 100 g of plant was extracted with 500 mL of water, ethanol, or acetone (Zorka, Sabac, Serbia) by heat reflux extraction, at a temperature of 90 °C and a duration of 2 h [36]. The mixtures were filtered through filter paper (Whatman, Maidstone, UK, No. 1). Dry extracts were obtained under reduced pressure at 45 °C, using a rotary evaporator (RV05 basic IKA, Staufen, Germany). The residues were stored in a dark glass bottle at +4 °C for further processing. The obtained residues were used for the chemical analysis.

### 4.2. Determination of the Total Phenolic (TPC) and Flavonoid (TFC) Contents

The Folin–Ciocalteu method was used for the determination of the TPC of extracts [9]. The reaction mixture was composed of 0.5 mL of extract (concentration 1 mg/mL), 5.0 mL of distilled water, and 0.5 mL of the Folin–Ciocalteu reagent (Merck, Darmstadt, Germany). After a period of 3 min, 1.0 mL of saturated sodium carbonate solution (Merck, Darmstadt, Germany) was added and the mixtures were shaken and kept in the dark for 1 h. The absorbance was measured at 725 nm using a microplate reader (Multiskan Spectrum, Thermo Corporation, Waltham, MA, USA). TPC was expressed as mg gallic acid (Merck, Darmstadt, Germany) equivalents per gram of dry extract (mg GA/g DE). The gallic acid calibration curve was plotted on the basis of seven calibration points within the range of 1.00–6.00 µg/mL of gallic acid in the reaction mixture. The TFC of the tested extracts was determined based on flavonoid affinity to form a complex with aluminum chloride (AlCl_3_) (Merck, Darmstadt, Germany). In fact, 0.5 mL of 2% AlCl_3_ and ethanol was mixed with the same volume of extracts (0.1–1.0 mg/mL). Absorption was measured at 415 nm after 1 h and ethanol was used as a blank. The total flavonoid content was calculated on the basis of the calibration curve of quercetin (Merck, Darmstadt, Germany) (0–50 mg/L). The mean of three readings was used and the TFC was expressed as milligrams of quercetin equivalents per gram of dry extract (mg QE/g of DE) [37].

### 4.3. LC–DAD–MS/MS Analysis of Extracts

Prepared extracts were diluted in mobile phase (0.05% aqueous formic acid—HCOOH and methanol MeOH, 1:1, *v*/*v*) to final concentrations of 2 mg/mL and analyzed by HPLC with LC–MS/MS detection. A series of dilutions of 35 reference standards (Sigma Aldrich, Burlington, MA, USA) were prepared in the 1.5 ng/mL to 25 μg/mL range, to perform quantification. Separation was achieved using a Series liquid chromatograph (Agilent Technologies 1200) coupled with a Triple Quad mass selective detector with electrospray ion source (Agilent Technologies 6410A, Santa Clara, CA, USA). Five microliters of extract/standard were injected and compounds were resolved on a Zorbax Eclipse XDB-C18 (50 × 4.6 mm, 1.8 μm) column, set at 50 °C. The mobile phase consisting of 0.05% HCOOH and MeOH, was delivered in gradient mode (0 min 30% B, 6 min 70%, 9–12 min 100%, and post time 3 min), at 1 mL/min flow. To verify the identity, this was followed by a UV/VIS signal in the range of 190–700 nm. After that, the effluent was passed to the MS/MS detector. Eluted compounds were detected in dynamic SRM (selected reactions monitoring) mode. The obtained results were analyzed using MassHunter Workstation Software Qualitative Analysis (B.06.00). A calibration curve (MRM peak area vs. concentration) was plotted for each compound [38,39].

### 4.4. Evaluation of Antioxidant Activity of Extracts

Investigated plant extracts were tested via the DPPH (1,1-diphenyl-2-picrylhydrazyl) assay using a spectrophotometric method adapted for microplates [13,40]. Ten microliters of the examined extract solutions, in a series of seven concentrations of double dilution (250–16,000 μg/mL for ethanol and acetone extracts and 125–4000 μg/mL for aqueous extracts as initial concentrations), were added to 100 μL of 90 μmol/L DPPH solution in methanol and the mixture was diluted with 190 μL of methanol (Merck, Darmstadt, Germany). The microplate reader measured absorption at 515 nm after 60 min (Multiskan Spectrum, Thermo Corporation). Synthetic antioxidant BHT (Merck, Darmstadt, Germany) served as a positive control. The radical-scavenging capacity (RSC) was calculated by the equation:

RSC % = (1 − A/(Acontrol − Asp)) × 100

Asp = the absorbance of the blind test and Acontrol = the absorbance of the DPPH radical (with no extract). The extract concentration that causes 50% DPPH inhibition (IC_50_) was calculated from the RSC concentration curve.

### 4.5. Preparation of Oral Liquid Formulation Loaded with YBE

Due to having the highest antioxidant potential, ethanol extract was selected for the preparation of the oral formulation and further examinations in animals. The composition of YBE oral liquid is presented in Table 3. YBE, xanthan gum (3 mg/mL) (Unichempharm, Cacak, Serbia), and mogroside (2 mg/mL) (Unichempharm, Cacak, Serbia) were precisely weighed, mixed together, dissolved in 100 °C distilled water, and continuously agitated until the formation of a dispersion. The obtained oral liquid contained YBE at a concentration of 31.25 mg/mL. The oral liquid formulation was stored in the fridge until the moment of application.

### 4.6. Characterization of Oral Formulation

#### 4.6.1. Determination of Organoleptic Properties

The organoleptic properties of the YBE such as color and odor were assessed 24 h after oral liquid preparation. Odor was determined by spreading formulation samples on a thin glass plate, while color was recorded by spreading a sample on a thin layer on a white paper to maximize color contrast. In order to be objective, three different investigators separately assessed these characteristics [41].

#### 4.6.2. Determination of the pH Values

The pH values of the prepared oral liquid were determined using a digital pH meter (Mettler Toledo, Columbus, OH, USA), which was calibrated before use with the standard buffer solution at pH 4.0, 7.0, and 9.0 (Mettler Toledo, Columbus, OH, USA). Measurements of pH value were carried out in triplicate [41]. The pH values were determined 24 h after oral liquid preparation.

#### 4.6.3. Determination of Microbiological Quality

The oral liquid formulation was subjected to microbiological tests in order to examine if it met the criteria for acceptable microbiological quality. The procedure for the microbiological control of non-sterile products involving count tests of microbes and specified microorganisms was performed according to previously established protocols. Microbial count refers to the average number of colony forming units (CFUs) found in agar. The prepared oral formulation was considered to meet specific microbiological standards if the total aerobic microbial count was less than 10^2^ CFU/mL, the total combined yeast/mold count less than 10^1^ CFU/mL, and it did not contain *Escherichia coli* [42].

### 4.7. Animals

Animals were treated in accordance with the Guide for the Care and Use of Laboratory Animals (8th edition, National Academies Press) and European Directive for the Welfare of Laboratory Animals No. 86/609/EEC and the principles of Good Laboratory Practice (GLP). The protocol of the current study was approved by the Ethics Committee for experimental animal well-being of the Faculty of Medical Sciences at the University of Kragujevac, Serbia.

This study was carried out using 20 spontaneously hypertensive *Wistar kyoto* rats (males, eight weeks old, body weight 200 ± 50 g). Animals were housed under controlled environmental conditions, at 22 ± 2 °C and a 12 h light/dark cycle, with ad libitum access to commercial rat food (20% protein rat food, Veterinary institute Subotica, Serbia) and tap water. The rats were randomly divided into the following groups:

- SHR—spontaneously hypertensive rats that received the base for the oral liquid formulation without YBE (*n* = 10);
- SHR + YBE—spontaneously hypertensive rats that received the oral liquid formulation by oral gavage (*n* = 10) (the applied volume provides 50 mg/kg of dry YBE per day).

The same procedure was repeated every day of the experimental protocol, for a duration of 4 weeks.

### 4.8. Assessment of the YBE Effect on Blood Pressure and Heart Rate

A day before sacrificing rats, blood pressure and heart rate were measured by a tail-cuff noninvasive method BP system (Rat Tail Cuff Method Blood Pressure Systems (MRBP-R), IITC Life Science Inc., Los Angeles, CA, USA) [43].

### 4.9. Assessment of the YBE Effect on Ex Vivo Rat Cardiac Function

A day after completing the 28-day drinking protocol, all of the animals were sacrificed. Short-term ketamine/xylazine-induced narcosis (a mixture of ketamine 50 mg/kg (100 mg/mL; Ketaset, Fort Dodge, IA, USA) and xylazine 10 mg/kg (100 mg/mL; AnaSed, Lloyd Laboratories, Shenandoah, IA, USA)) was used, with decapitation afterwards. The chest was then opened via midline thoracotomy. The hearts were immediately removed and immersed in cold saline and mounted on a stainless-steel cannula on the Langendorff perfusion apparatus that provided retrograde perfusion under constant coronary perfusion pressure CPP = 70 cmH_2_O. Krebs–Henseleit buffer was used for retrograde perfusion (in mmol/L: NaCl 118, KCl 4.7, CaCl_2_·2H_2_O 2.5, MgSO_4_·7H_2_O 1.7, NaHCO_3_ 25, KH_2_PO_4_ 1.2, glucose 11, and pyruvate 2) (Merck, Darmstadt, Germany). The buffer was equilibrated with 95% O_2_ and 5% CO_2_, with a pH of 7.4 and a temperature of 37 °C. After placing the sensor in the left ventricle, the parameters of myocardial function have been continuously measured: the maximum rate of pressure development in the left ventricle (dp/dt max), minimum rate of pressure development in the left ventricle (dp/dt min), systolic left ventricular pressure (SLVP), diastolic left ventricular pressure (DLVP), and heart rate (HR). Coronary flow (CF) was measured flowmetrically [13].

After the establishment of heart perfusion, the hearts were stabilized within 30 min. In all groups, after a stabilization period, hearts were subjected to a 20 min period of global ischemia (perfusion was totally stopped), followed by a 30 min period of reperfusion. In the reperfusion period, we measured all cardiodynamic parameters and coronary flow in 5 min intervals. The following data points such as S—stabilization, R1—the first minute of reperfusion, and R7—the last minute of reperfusion were used for statistical analysis.

### 4.10. Assessment of the YBE Effects on Systemic Redox State

After sacrificing, blood samples for biochemical analysis were collected from a jugular vein at the end of the treatment with YBE in order to test the systemic redox state. After the centrifugation of heparinized venous blood, plasma and erythrocytes were separated. In plasma, the following prooxidants were determined: the levels of superoxide anion radical (O_2_^−^), nitrites (NO_2_^−^), hydrogen peroxide (H_2_O_2_), and the index of lipid peroxidation (measured as thiobarbituric acid reactive substances (TBARS)). Parameters of the antioxidative defense system such as the activities of superoxide dismutase (SOD) and catalase (CAT) and level of reduced glutathione (GSH) were determined in erythrocyte samples [13].

### 4.11. Histological Analysis

Hematoxylin–eosin staining (HE) method was carried out in order to evaluate the effects of YBE liquid formulation on the cell morphology of the myocardium, kidney, and liver in accordance with a previously reported method [13]. The heart and kidney of rats were assessed via scoring systems while liver changes were expressed by a descriptive method. The degree of tissue damage was estimated according to a previously described scale which classifies organ damage by using grades 0–IV (Table 4) [10,44]. Renal damage assessment was performed based on the presence of the following characteristics, according to the defined scale (Table 4) [44]:

1. Loss of brush cover (tubule epithelium simplification);
2. Loss of epithelial cells;
3. Necrosis;
4. Intraluminal cell debris;
5. Intraluminal homogeneous cylinders.

### 4.12. Statistical Analysis

IBM SPSS Statistics 23.0 for Windows was used for the statistical analysis of the antioxidant activity of the extracts as well as for data within the SHR and SHR + YBE group. Three measured points were statistically analyzed: the first point was stabilization (S) and the second and third points were the first and the last point of the 30 min reperfusion period (R1 and R7). Values were expressed as mean ± standard deviation (SD). Data distribution was checked by the Shapiro–Wilk test. Data were analyzed using a one-way analysis of variance (ANOVA) and the post hoc Bonferroni test for multiple comparisons. Values of *p* < 0.05 were considered to be statistically significant, while values of *p* < 0.01 were considered to be highly statistically significant.

## 5. Conclusions

Based on the findings of the current study, the yellow bedstraw-based oral liquid formulation that was developed has the potential to reduce blood pressure values and preserve cardiac function in hypertensive conditions. This formulation represents an effective and safe natural approach for the alleviation of hypertension and associated cardiovascular complications that can be of considerable interest for the pharmaceutical industry. However, further clinical studies are necessary in order to fully clarify the potential of a yellow bedstraw oral liquid formulation before implementation as an additive strategy for hypertensive management.

## Figures and Tables

**Table 1:** Quantitative and qualitative analysis of individual compounds found in aerial part of yellow bedstraw, expressed as µg/g of dry extract.

Name of Compound	YBE-AE	YBE-E	YBE-AqE	Retention Time [t_R_]
Ursolic acid	5376 ± 35	8900 ± 58	30.3 ± 0.2	9.5
Chlorogenic acid	20,368 ± 1018	15,561 ± 778	27,420 ± 1371	0.8
Isoquercetin	6900 ± 207	6873 ± 206	290 ± 8.7	2.25
Cynaroside	6918 ± 207	9612 ± 288	5032 ± 151	2.13
Quercetin	145.6 ± 43.7	179.1 ± 53.7	12.2 ± 3.7	3.74
p-Coumaric acid	44.9 ± 4.0	54.3 ± 4.9	137.6 ± 12.4	1.69
Astragalin	72.4 ± 2.9	93.8 ± 3.8	63.7 ± 2.5	2.8
Caffeic acid	24.2± 1.7	19.6 ± 1.4	22.6 ± 1.6	1.18
Ferulic acid	15.8 ± 1.6	17.3 ± 1.7	38.8 ± 3.9	1.9
Luteolin	19.7 ± 1.0	33.9 ± 1.7	10.0 ± 0.5	4.03
Isorhamnetin	38.5 ± 2.3	60.2 ± 3.6	2.2 ± 0.1	4.79
Apigetrin	22.5 ± 1.1	45.8 ± 2.3	15.5 ± 0.8	2.81
Vanillic acid	15.9 ± 4.8	14.7 ± 4.4	14.5 ± 4.4	1.24
*p*-Hydroxybenzoic acid	10.6 ± 0.6	8.0 ± 0.5	26.7 ± 1.6	1.08
Kaempferol	9.4 ± 0.6	7.3 ± 0.5	0.5 ± 0.04	4.55
Hesperetin	12.9	14.4	1.0	4.44
Protocatechuic acid	1.9 ± 0.2	1.8 ± 0.1	3.1 ± 0.2	0.79
Rhamnetin	<5.28	<4.89	<4.89	5.84
Diosmetin	6.1	12.4	2.9	5.02
Rosmarinic acid	1.9	0.3	1.4	2.43
Alizarin	<2.6	<2.44	<2.44	5.95
Epicatechin	<0.65	<0.61	<0.61	0.95
Glycyrrhizin	<0.16	<0.16	<0.16	7.41
Hyperoside	<0.32	<0.31	<0.31	2.16
Catechin	<0.65	<0.61	<0.61	0.74
Myricetin	<20.85	<19.55	<19.55	2.67

YBE-AE—yellow bedstraw acetone extract; YBE-E—yellow bedstraw ethanol extract; and YBE-AqE—yellow bedstraw aqueous extract.

**Table 2:** Blood pressure and heart rate values in treated and untreated spontaneously hypertensive rats.

Parameter	SHR	SHR + YBE
SBP	185.2 ± 5.6	155.3 ± 7.8 **
DBP	105.1 ± 11.1	85.5 ± 5.1 *
HR	420.2 ± 25.3	390.7 ± 17.9

SBP—systolic blood pressure; DBP—diastolic blood pressure; HR—heart rate; SHR—spontaneously hypertensive rats; and YBE—yellow bedstraw extract. Values are expressed as mean ± standard deviation (SD). *, statistical significance at the level of *p* < 0.05 in relation to SHR group; **, statistical significance at the level of *p* < 0.01 in relation to SHR group; and *n* = 10 per group. Data were analyzed using an independent samples *t*-test.

**Table 3:** Components and quantity of oral liquid formulation with YBE.

Formulation	Components	Quantity (g)
YBE oral Liquid Formulation	YBE	3.1
	Xanthan gum	0.3
	Mogroside	0.2
	Purified water	ad 100

YBE—yellow bedstraw extract.

**Table 4:** Scale for evaluation of the extent of changes in the heart and kidney structure.

Degree of Changes	Myocardial Tissue Damage	Percentage of Tubular Damage
Grade 0	Regular histomorphology, discrete degenerative changes	<10%
Grade I	Focal and unicellular necrosis limited to the subendocardial band, discrete degenerative changes	10–25%
Grade II	Focal, spotty necrosis	26–50%
Grade III	Larger fields of necrosis	51–75%
Grade IV	Extensive fields of confluent necrosis	>75%

## Data Availability

The authors confirm that the data supporting the findings of this study are available within the article.
